# Intravitreal Voriconazole for the Treatment of *Cryptococcus neoformans* Endogenous Endophthalmitis

**DOI:** 10.31486/toj.19.0043

**Published:** 2020

**Authors:** Asghar A. Haider, J. R. Gallagher, Jordan S. Johnson, Joseph D. Benevento

**Affiliations:** ^1^ Department of Ophthalmology, Louisiana State University Health Sciences Center, New Orleans, LA; ^2^ Department of Ophthalmology, Ochsner Clinic Foundation, New Orleans, LA

**Keywords:** *Cryptococcus*, *Cryptococcus neoformans*, *endophthalmitis*, *eye infections–fungal*, *intravitreal injections*, *voriconazole*

## Abstract

**Background:** *Cryptococcus neoformans* is an encapsulated yeast that can cause fungemia and, in rare instances, lead to endogenous fungal endophthalmitis. No standard of care has been established to treat fungal endophthalmitis when systemic antifungal treatment fails to resolve the intraocular infection. Intravitreal voriconazole has been used for the treatment of fungal endophthalmitis caused by a broad range of fungal pathogens, and a limited number of reports have shown the efficacy of using intravitreal voriconazole for *C neoformans* endophthalmitis. We report a case of endogenous fungal endophthalmitis caused by *C neoformans* that was responsive to intravitreal voriconazole.

**Case Report:** A previously healthy 57-year-old male diagnosed with primary neuroendocrine lung tumor developed endogenous endophthalmitis from *C neoformans*. The endophthalmitis was resistant to intravenous amphotericin B treatment but was responsive to intravenous fluconazole in one eye and was apparently more responsive to intravitreal voriconazole in the other eye.

**Conclusion:** Intravitreal voriconazole should be considered for the treatment of cryptococcal endophthalmitis.

## INTRODUCTION

*Cryptococcus neoformans* is an encapsulated yeast commonly found in soil.^1^ Several different species can cause cryptococcal disease, and based on a 2015 phylogenetic analysis, 7 distinct species have been identified, with the most common pathogen being *C neoformans*.^2^ Infection from *C neoformans* usually presents as pneumonia and spreads hematogenously to other organs. Ocular involvement usually occurs after cryptococcal meningitis from hematogenous dissemination through the leptomeninges. Intraocular manifestation includes vitritis, choroiditis, exudative retinal detachment, neuroretinitis, and endophthalmitis.^1,3^ Cryptococcal endophthalmitis is an extremely rare form of fungal endophthalmitis and is typically treated with intravenous (IV) amphotericin B.^4-6^ Delayed diagnosis and lack of standardized treatment protocols make fungal endophthalmitis challenging to treat, and few cases of successful treatment of cryptococcal endophthalmitis have been reported.^7^

Patients with fungal endophthalmitis usually have multiple predisposing systemic conditions, including recent hospitalization, diabetes mellitus, liver disease, renal failure, cancer, indwelling lines, systemic surgery, organ transplantation, human immunodeficiency virus/acquired immunodeficiency syndrome, IV drug use, and immunosuppressive therapy, although cases of healthy immunocompetent patients have also been reported.^3,8^

When traditional systemic antifungal treatment is inadequate, alternative approaches such as newer classes of antifungal drugs, vitrectomy, and intravitreal injection have shown promise.^9^ We report a case of endogenous fungal endophthalmitis caused by *C neoformans* that was responsive to intravitreal voriconazole.

## CASE REPORT

A 57-year-old previously healthy male was found to have biopsy-proven primary right lung neuroendocrine tumor while being treated for pneumonia. During his malignancy evaluation, he developed altered mental status and experienced multiple cerebral embolic vascular accidents. No source of emboli was found with either transesophageal or transthoracic echocardiogram. The etiology was postulated to be the hypercoagulable state from the underlying malignancy. The plan was surgical removal of the tumor; however, during the patient's hospital stay, his clinical status declined, and he required ventilator support to maintain his oxygen saturation. He was transferred to our tertiary care center for further workup and treatment.

Magnetic resonance imaging of the brain was concerning for leptomeningeal inflammation and hydrocephalus. Lumbar puncture revealed cryptococcal antigen, and induction therapy with amphotericin B 5 mg/kg IV every 24 hours and flucytosine 25 mg/kg IV every 6 hours for 4 weeks was begun for the treatment of cryptococcal meningitis. The patient also received ampicillin 2 g IV every 4 hours, ceftriaxone 2 g IV every 12 hours, and vancomycin 1,500 mg once, followed by 1,000 mg every 12 hours. An external ventricular drain was placed to monitor intracranial pressure (ICP). After multiple lumbar punctures with elevated opening pressures and concern for uncontrolled ICP, a ventriculoperitoneal shunt (VPS) was placed.

**Figure 1.**
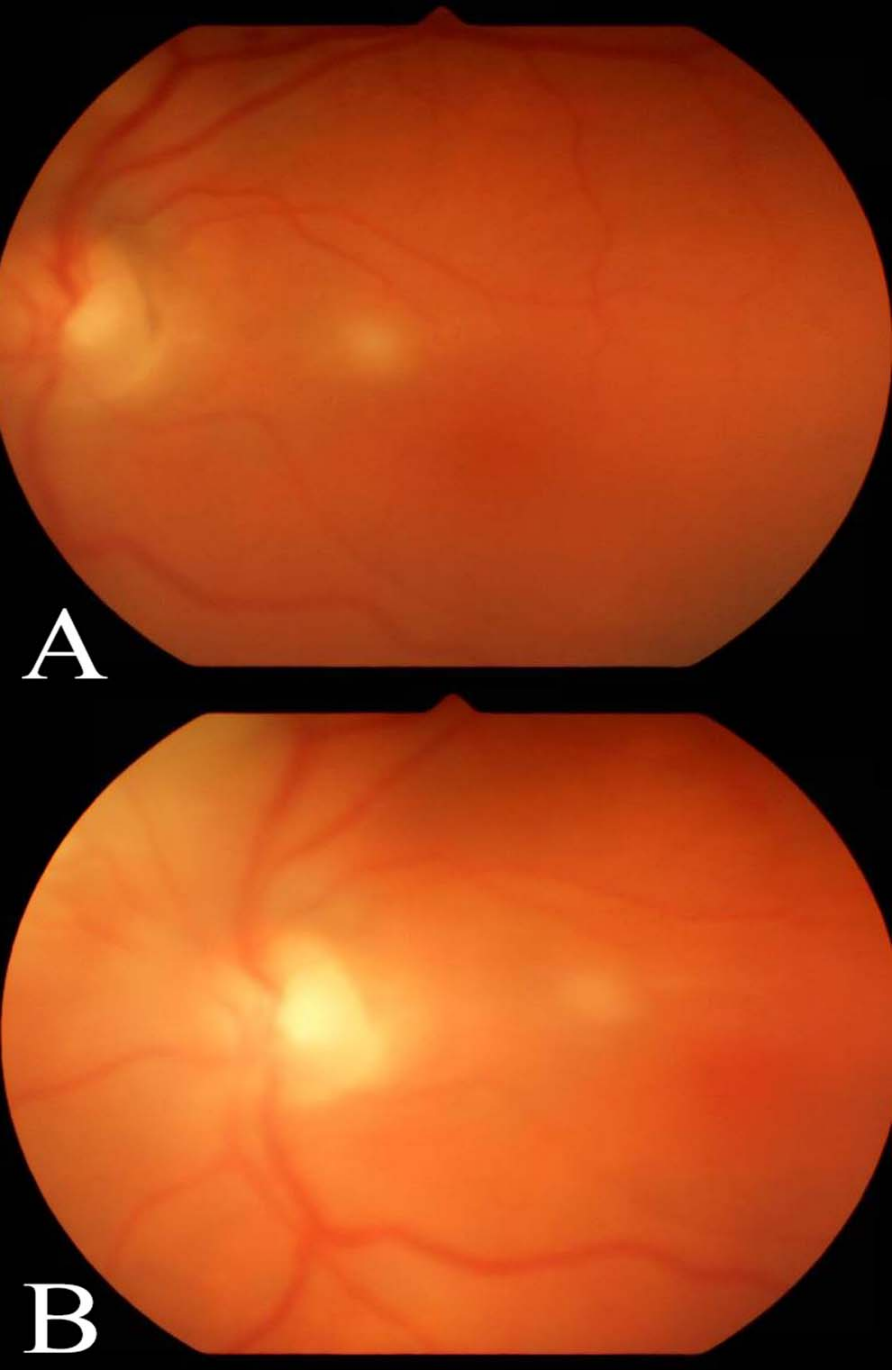
**(A) Fundus photograph of the left eye from the initial dilated examination demonstrates a macular lesion with overlying vitritis. (B) Fundus photograph 4 weeks after initial evaluation shows regression of lesion.**

**Figure 2.**
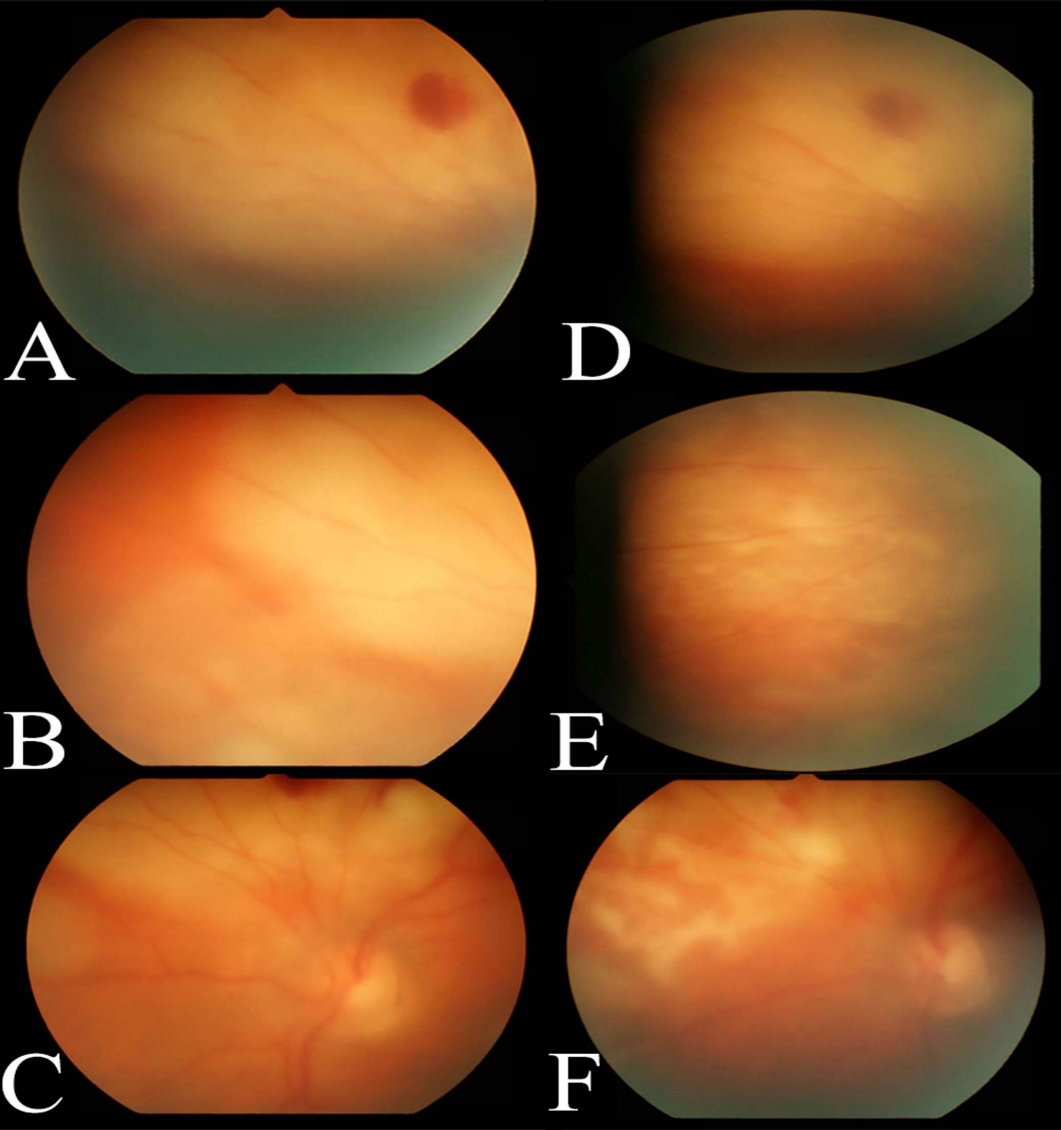
**Fundus photographs of the left eye from the initial evaluation show a peripapillary lesion (A) with white chorioretinal infiltrates with vitritis (B and C). The peripapillary lesion showed worsening vitritis indicating progression at 1 week (D). Vitritis and chorioretinal infiltrates showed regression at the subsequent 2-week (E) and 4-week visits (F).**

**Figure 3.**
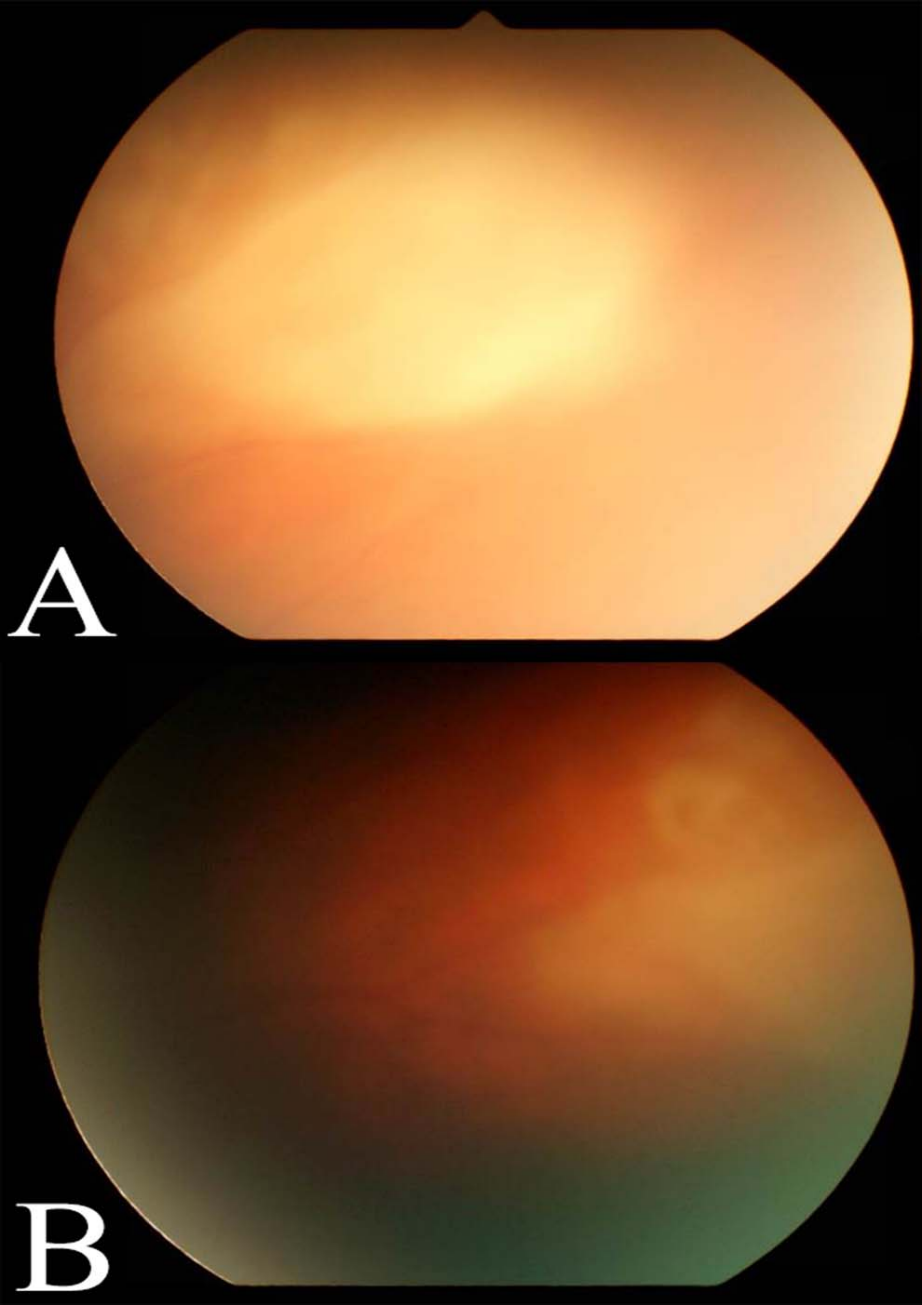
**Fundus photograph of the right eye 1 week after treatment (A) demonstrates a large chorioretinal infiltrate with overlying vitritis. The lesion responded well to treatment with a markedly regressed appearance at 2-week follow-up (B).**

Ophthalmology was consulted approximately 1 month after the patient was transferred. Dilated fundus examination revealed bilateral multifocal, elevated, white chorioretinal infiltrates with vitritis. The left eye had a macular lesion (Figure 1A) and a peripapillary lesion that was more elevated and appeared to penetrate more into the vitreous than the other lesions (Figures 2A, 2B, and 2C). The right eye had a non–macula-threatening infiltrate (Figure 3A). At that time, the systemic amphotericin B was changed to fluconazole 800 mg IV every 12 hours.

Repeat dilated examination of the left eye showed progression of the peripapillary lesion (Figure 2D), prompting a vitreous tap and intravitreal injection of 0.1 mL voriconazole (100 μg/0.1 mL). The patient's vitreous cultures were negative, and serial examinations revealed initial stability followed by gradual improvement (Figures 2E and 2F). One week later, the intravitreal injection of voriconazole was repeated in the left eye because of the persistence of possible early retina breakthrough of the nasal infiltrate. The infiltrate in the right eye followed a nonprogressive course and thus was not treated with intravitreal injections. The infiltrates in the left eye showed regression, with early chorioretinal atrophic scarring around fading infiltrates (Figures 1B, 2E, and 2F). The lesion in the right eye (Figure 3B) followed the same course. Complete resolution was not documented because the patient transferred to an out-of-state specialty center.

## DISCUSSION

*C neoformans* causing endogenous endophthalmitis is rare, and the exact incidence is unknown.^10^ *Candida* species cause the majority of reported cases of fungal endophthalmitis, and most occur in immunocompromised hosts.^3,5,11,12^ The literature provides limited guidance for the treatment of fungal endophthalmitis caused by other species such as *C neoformans.* Effective treatment is further challenged by the difficulty of early diagnosis and the ocular efficacy of commonly used systemic antifungals.

Our patient had several risk factors for developing endogenous fungal endophthalmitis, including recent hospitalization, cancer, respiratory disease, and presence of an IV line.^3^ Additionally, according to 2 recent (2018 and 2016) reports, our patient's VPS may have also predisposed him to cryptococcal infection.^7,13^ Of these 2 reported cases of VPS-associated *Cryptococcus*, 1 patient did not have ocular involvement, and the symptoms rapidly resolved with the removal of VPS and administration of oral flucytosine and IV amphotericin B.^13^ The patient in the case reporting fungal endophthalmitis associated with a VPS had a poor response to amphotericin B, but the lesion was stabilized with systemic fluconazole.^7^ These cases highlight the potential link between cryptococcal infection and VPS.

Our patient underwent an ophthalmic evaluation after he was noted to have ventilator-related conjunctival chemosis. Altered mental status and significant comorbidities prevented our patient from reporting changes in visual acuity or pain with eye movement, potentially delaying more timely ophthalmology consultation.

As stated previously, initial treatment for endogenous fungal endophthalmitis usually involves systemic amphotericin B.^4^ The many well-studied disadvantages of using systemic amphotericin B include questionable ocular penetration, poor side effect profile, and retinal toxicity.^14^ The lack of consensus for an alternative treatment usually means that other antifungals are only used when initial treatment is ineffective, possibly causing an unnecessary delay in optimal treatment.

Newer antifungals are available and have shown efficacy in the treatment of fungal endophthalmitis.^4,5,15^ Antifungal susceptibility studies have shown that an increasing number of clinical isolates are more susceptible to voriconazole and other antifungal agents compared to amphotericin B alone.^6,16^ Our patient received amphotericin B and flucytosine, which were sufficient to stabilize the lesions in the right eye. However, intravitreal voriconazole was used for the persistent lesions in the left eye. This clinical course supports the use of systemic antifungal therapy in fungal endophthalmitis, especially when the disease is bilateral, but also highlights the importance of combination therapy and the use of intravitreal antifungal therapy for more aggressive disease. This strategy of combination therapy with multiple antifungal agents has been advocated by other authors for treatment of cryptococcosis.^6,15^

Of note, our patient did receive a long course of combination systemic antifungal medications, and this treatment may explain why no organism was isolated from the vitreous fluid biopsy and blood cultures. Treatment response assessment was based on his clinical examination that showed a significant improvement in vitreoretinal lesions after intravitreal injection in the left eye.

## CONCLUSION

Intravitreal voriconazole as primary treatment or in combination with systemic antifungal medication should be considered in patients with *C neoformans* fungal endophthalmitis. Studies are needed to evaluate dosing, optimal combination therapy, and appropriate follow-up intervals, but given the rarity of cryptococcal fungal endophthalmitis, a multicenter collaboration would likely be necessary.

## Notes

This article meets the Accreditation Council for Graduate Medical Education and the American Board of Medical Specialties Maintenance of Certification competencies for Patient Care and Medical Knowledge.
